# Influence of cannabis use on incidence of psychosis in people at clinical high risk

**DOI:** 10.1111/pcn.13555

**Published:** 2023-05-13

**Authors:** Lucy A. Chester, Lucia R. Valmaggia, Matthew J. Kempton, Edward Chesney, Dominic Oliver, Emily P. Hedges, Elise Klatsa, Daniel Stahl, Mark van der Gaag, Lieuwe de Haan, Barnaby Nelson, Patrick McGorry, G. Paul Amminger, Anita Riecher-Rössler, Erich Studerus, Rodrigo Bressan, Neus Barrantes-Vidal, Marie-Odile Krebs, Birte Glenthøj, Merete Nordentoft, Stephan Ruhrmann, Gabriele Sachs, Philip McGuire

**Affiliations:** 1 Department of Psychosis Studies, Institute of Psychiatry, Psychology & Neuroscience, King’s College London, London, UK; 2 Department of Psychology, Institute of Psychiatry, Psychology & Neuroscience, King’s College London, London, UK; 3 Department of Psychiatry, Oxford University, Warneford Hospital, Oxford, UK; 4 Department of Forensic and Neurodevelopmental Sciences, Institute of Psychiatry, Psychology & Neuroscience, King’s College London, London, UK; 5 Department of Biostatistics, Institute of Psychiatry, Psychology & Neuroscience, King’s College London, London, UK; 6 Faculty of Behavioural and Movement Sciences, Department of Clinical Psychology and EMGO+ Institute for Health and Care Research, VU University, Amsterdam, The Netherlands; 7 Department of Psychosis Research, Parnassia Psychiatric Institute, The Hague, The Netherlands; 8 Department Early Psychosis, Amsterdam UMC, Amsterdam, The Netherlands; 9 Arkin Amsterdam, Amsterdam, The Netherlands; 10 Centre for Youth Mental Health, University of Melbourne, Parkville, Victoria, Australia; 11 Orygen, Parkville, Victoria, Australia; 12 Faculty of Medicine, University of Basel, Basel, Switzerland; 13 Department of Psychology, Division of Personality and Developmental Psychology, University of Basel, Basel, Switzerland; 14 LiNC—Lab Interdisciplinar Neurociências Clínicas, Depto Psiquiatria, Escola Paulista de Medicina, Universidade Federal de São Paulo – UNIFESP, Sao Paulo, Brazil; 15 Departament de Psicologia Clínica i de la Salut, Universitat Autònoma de Barcelona, Fundació, Sanitària Sant Pere Claver (Spain), Spanish Mental Health Research Network (CIBERSAM), Barcelona, Spain; 16 Hôpital Sainte-Anne, C’JAAD, Service Hospitalo-Universitaire, Inserm U894, Institut de Psychiatrie (CNRS 3557), University Paris Descartes, Paris, France; 17 Centre for Neuropsychiatric Schizophrenia Research (CNSR) & Centre for Clinical Intervention and Neuropsychiatric Schizophrenia Research (CINS), Mental Health Centre Glostrup, University of Copenhagen, Glostrup, Denmark; 18 Mental Health Center Copenhagen and Center for Clinical Intervention and Neuropsychiatric Schizophrenia Research, CINS, Mental Health Center Glostrup, Mental Health Services in the Capital Region of Copenhagen, University of Copenhagen, Kobenhavn, Denmark; 19 Department of Psychiatry and Psychotherapy, Faculty of Medicine and University Hospital, University of Cologne, Cologne, Germany; 20 Department of Psychiatry and Psychotherapy, Medical University of Vienna, Wien, Austria

**Keywords:** clinical high-risk, longitudinal, psychotic disorders, substance use, THC

## Abstract

**Aims:**

Evidence for case–control studies suggests that cannabis use is a risk factor for the development of psychosis. However, there have been limited prospective studies and the direction of this association remains controversial. The primary aim of the present study was to examine the association between cannabis use and the incidence of psychotic disorders in people at clinical high risk of psychosis. Secondary aims were to assess associations between cannabis use and the persistence of psychotic symptoms, and with functional outcome.

**Methods:**

Current and previous cannabis use were assessed in individuals at clinical high risk of psychosis (*n* = 334) and healthy controls (*n* = 67), using a modified version of the Cannabis Experience Questionnaire. Participants were assessed at baseline and followed up for 2 years. Transition to psychosis and persistence of psychotic symptoms were assessed using the Comprehensive Assessment of At-Risk Mental States criteria. Level of functioning at follow up was assessed using the Global Assessment of Functioning disability scale.

**Results:**

During follow up, 16.2% of the clinical high-risk sample developed psychosis. Of those who did not become psychotic, 51.4% had persistent symptoms and 48.6% were in remission. There was no significant association between any measure of cannabis use at baseline and either transition to psychosis, the persistence of symptoms, or functional outcome.

**Conclusions:**

These findings contrast with epidemiological data that suggest that cannabis use increases the risk of psychotic disorder.

There is a considerable body of evidence linking cannabis use with an increased risk of developing a psychotic disorder. Cannabis use is more common in patients with psychosis than in the general population,^1–3^ and the risk may be higher if use begins in adolescence,^4–6^ is frequent,^7–10^ and involves cannabis with a high d-9 tetrahydrocannabinol (THC) content.^2,6,11^ However, the direction of this association remains controversial^12^: the presence of a psychotic disorder may increase the likelihood of cannabis use,^13^ patients with psychotic disorders use cannabis to relieve psychotic symptoms,^14–16^ and genetic factors that increase the likelihood of cannabis use may be more common in patients with psychosis than the general population.^17,18^ Much of the data relating cannabis use to psychosis have been derived from interviewing patients after they have developed a psychotic disorder.^2,7,11^ These data thus reflect patients’ retrospective assessments of their premorbid cannabis use, and recall accuracy may be influenced by the effects of time and of the disorder.^19^ Only a few prospective studies have examined cannabis use and the incidence of psychosis in general population samples, although these have found some associations between cannabis use and the later onset of psychosis, the large scale of these studies (which involved thousands of participants) precluded a detailed assessment of cannabis use.^5,20,21^

The Clinical High-Risk (CHR) state is a clinical syndrome that typically occurs in adolescents and young adults. It is associated with a very high risk of developing a psychotic disorder, with around 19% of CHR individuals becoming psychotic within 2 years of presentation.^22^ To date, only a limited number of studies have investigated the relationship between cannabis use in CHR individuals and the subsequent incidence of psychosis, and the findings have been inconsistent. A recent meta-analysis^23^ did not find a significant difference in risk of transition to psychosis between CHR cannabis users and non-users, but highlighted the need to assess cannabis use in more detail. Further meta-analytical results suggest that while lifetime use of cannabis is not significantly associated with transition rates, the relative risk is greater in those with cannabis abuse or dependence, likely a marker for heaver cannabis use.^24^ Results from the few studies which have specifically measured frequency of cannabis use and age of first use have been mixed,^25–27^ with only Valmaggia *et al*.^25^ finding a signifi-cant association with risk of psychosis.

The primary aim of the present study was to examine the association between cannabis use and the incidence of psychosis in people at clinical high risk. Secondary aims were to assess associations between cannabis use and the persistence of psychotic symptoms, and with functional outcome. In a prospective design, cannabis use was comprehensively assessed in a large sample of CHR subjects that was then followed for 2 years to determine clinical outcomes. Based on the previous literature in CHR subjects, we hypothesized that neither current nor previous cannabis use *versus* non-use would be associated with an increased incidence of later psychosis, but that a high frequency of cannabis use, use before the age of 16, the use of high potency (>10% THC) cannabis strains, and current cannabis dependence would be. Secondary hypotheses were that cannabis use would be linked with non-remittance from the CHR state (persistence of symptoms) and a poor functional outcome.

## Methods

### Recruitment of participants

Participants were recruited to a multi-centre prospective study of people at CHR for psychosis.^28^ Three hundred forty-four CHR participants meeting Comprehensive Assessment of At-Risk Mental States (CAARMS) criteria^29^ for an ultra-high risk state were enrolled from 11 centers in Europe, Australia and South America. Sixty-seven healthy controls (HCs) were recruited from four of the sites: London, Amsterdam, Den Haag, and Melbourne. The HC sample matched (at group level) the CHR sample in terms of age and gender.

### Inclusion and exclusion criteria

The study guidelines recommended that participants should be 16–35 years old. While most of the sample (95.0%) was in this age range, a few sites included individuals who were slightly older (*n* = 3) or younger (*n* = 14) than this range as the local clinical services for CHR subjects used a slightly broader age range. Exclusion criteria were: previous diagnosis of a psychotic disorder, as defined by the Structural Clinical Interview for DSM Disorders^30^; exceeding the ‘Psychosis Threshold’ or ‘Antipsychotic Treatment Threshold’, defined by the CAARMS^29^; an estimated IQ < 60 as measured by the shortened WAIS^31^; being unwilling to give a blood or saliva sample for genetic analysis. In addition, CHR subjects were excluded if their psychotic symptoms could be explained by an organic disorder or substance misuse, and HC were excluded if they met CAARMS criteria for the CHR state. Written, informed consent was provided by all participants.

### Ethics statement

The study protocol was approved by the relevant research ethics committees at each study site. All procedures conductive to the present work are in compliance with the ethical standards of the relevant national and institutional committees on human experimentation and with the Helsinki Declaration of 1975 as revised in 2008.

### Baseline assessments

Cannabis use was assessed using a modified form of the Cannabis Experience Questionnaire (EU-GEI_CEQ_).^7^ Participants were first asked if they had ever used cannabis. If the answer was yes, they were asked if they were a current or an ex-user, and to describe their typical pattern of use. Age at first cannabis use was estimated by the participant, with collateral information from informants if available. The presence of cannabis dependence in the year prior to baseline was assessed using DSM-IV criteria for substance dependence.^32^ Participants were also asked to describe the type of cannabis that they used the most. This description was used by the investigators to classify the cannabis used as having either a high (>10%) or low (<10%) THC content, using data published by the European Monitoring Centre for Drugs and Drug Addiction 2016 report^33^ and national data reports^34–52^ (see Supplementary Materials, Table S1).

Global functioning was assessed using the Global Assessment of Functioning (GAF) disability subscale.^53^ Use of tobacco and alcohol were recorded using the Composite International Diagnostic Interview.^54^ Use of other recreational drugs were collected using the EU-GEI_CEQ_. Sociodemographic data were collected using the Medical Research Council Sociodemographic Schedule.^55^

### Assessment of clinical outcomes

Participants had face-to-face assessments at baseline, 12 and 24 months. When a CHR individual developed psychosis, a follow-up assessment was conducted as close to psychosis onset as possible. The primary outcome was transition to psychosis within 2 years, defined according to CAARMS criteria.^29^ Secondary outcomes included persistence of symptoms, defined as still meeting CAARMS criteria for the CHR state or having transitioned to a psychotic disorder, and level of functioning at the latest available follow up timepoint.

### Statistical analysis

CHR participants for whom there were no cannabis use data (*n* = 10) were excluded from analysis. Differences between the CHR and HC groups were assessed using either independent *t*-tests or ANOVA models for continuous data, and either Pearson’s chi squared test or Fisher’s exact test for categorical data.

Cannabis use variables were coded as follows: Cannabis use status – 0 = never used, 1 = past user, 2 = current user; Age of first cannabis use – 0 = aged 16 years or older, 1 = aged 15 years or younger; Frequency of cannabis use – 0 = less than once weekly, 1 = more than once weekly/less than daily, 2 = daily; THC content of most used cannabis type – 0 = less than 10% THC, 1 = more than 10% THC; Cannabis dependence – 0 = no cannabis dependence in past 12 months, 1 = cannabis dependent in past 12 months. Participants who had never used cannabis were excluded from the age of first use, frequency of use, THC content and cannabis dependence variables, such that cannabis users were compared with each other.

For the primary outcome, we completed survival analyses with the outcome of time to psychosis onset, with outcomes censored at 2 years post baseline. Kaplan–Meier survival curves for each cannabis predictor variable, without covariates, were inspected to assess for proportional hazards. Variables which met our threshold (*P* < 0.2) for univariate analyses were included in multilevel Cox regression analyses, using the coxme package for R. Site was included as a random effect to account for clustering. Effect sizes were quantified as hazard ratios (HR) and 95% confidence intervals.

For persistence of symptoms, cannabis variables which met our threshold (*P* < 0.2) in chi-square or Fisher’s exact test analyses were input into multilevel logistic regression models using the lme4 package for R. Site was included as a random effect. Effect sizes for the remission outcome were quantified as odds ratios (ORs) with 95% confidence intervals.

For functional outcome, we used Spearman Rank Correlation and *t*-tests with the outcome of GAF score at the latest follow-up assessment. Cannabis variables which met our threshold (*P* < 0.2) in univariate analyses were input into multilevel linear regression models using the lme4 package for R. Time (in days) from baseline to the last GAF assessment was added as a covariate to account for possible deviation around the planned assessment date. Site was included as a random effect. To analyze the difference between the mean change scores of GAF from baseline to follow-up, baseline GAF score was added as a covariate to the multilevel models. Fixed effect parameter estimates were quantified with 95% confidence intervals (see Supplementary Materials for interpretation).

Potential confounders were identified from recent meta-analyses,^56–58^ and included age, gender, ethnicity, tobacco, alcohol, and other substance use. Potential confounders were not included as *a priori* defined covariates in all analyses to prevent overfitting. Instead, confounding variables which met our threshold (*P* < 0.2) in univariate analyses were included in sensitivity analyses. Potential confounders were added to each multilevel model in a forward stepwise fashion and the maximum log likelihood of the new and old models was compared. Confounders which significantly improved the model were retained, and the process was repeated with the next confounder.

All analyses were performed using R version 4.0.3 and SPSS version 25. Statistical significance was defined at the 0.05 level.

## Results

### Comparison of CHR and HC populations

At baseline, there were no differences in the age, gender, or ethnicity of the CHR and HC groups, but the former were more likely to use tobacco (Table 1). 9.3% of the CHR group were taking an antipsychotic medication. CHR participants were more likely to have ever used cannabis, to use cannabis frequently, and to use high potency cannabis (Table 1). When these comparisons were repeated after restricting the CHR sample to participants recruited from the sites that had also recruited HC, these findings were unchanged (Table S2).

### Cannabis use and clinical outcomes

There were no socio-demographic differences between CHR participants who completed follow-up and those with missing follow-up data (Table S3). 248 (74.3%) of CHR participants had ever used cannabis, of whom 90 (26.9%) were current users at baseline. Cannabis users were on average older than non-users (past users +2.4 years, current users +2.8 years) and used more tobacco products, alcohol, and other substances. Current cannabis users were more likely to be male than non-users, and used more tobacco and other substances than past users (Table S4).

### Onset of psychosis

62 (18.6%) of 334 CHR participants developed psychosis during follow up. The mean time to transition was 380 days (SD = 411.6), with an interquartile range of 121–496 days (Fig. S1). There were no significant differences in demographic or clinical features between subjects who did or did not subsequently develop psychosis (Table S5), save that more of the former were taking antipsychotic medications at baseline (HR 2.375 [95% CI: 1.185–4.758], *P* = 0.015).

**Figure 1:**
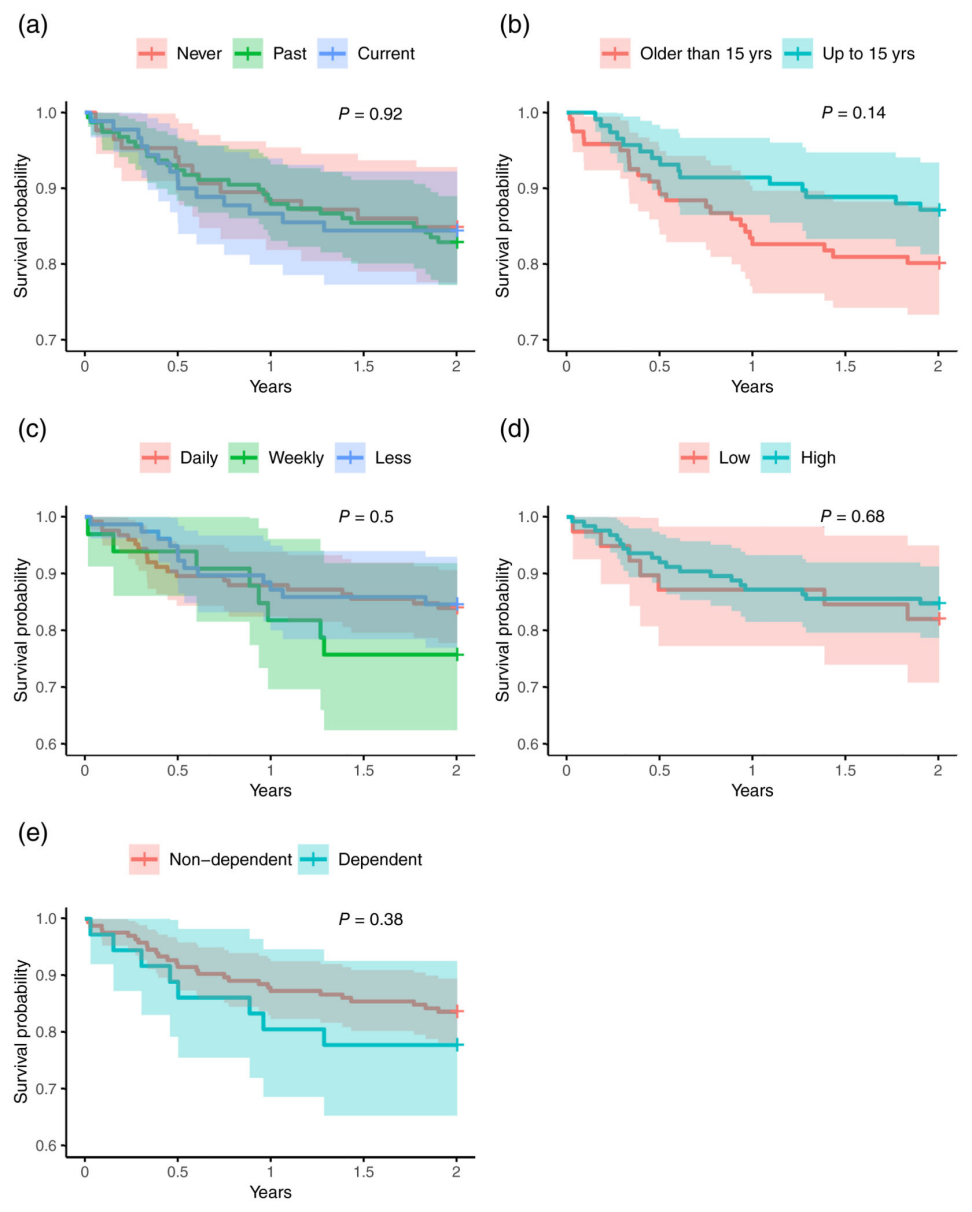
Kaplan-Meier survival curves showing relationship between cannabis use at baseline in the CHR group and transition to psychosis. (a) Current-user/ Ex-user/Never used. (b) Age first used cannabis. (c) Cannabis frequency. (d) Cannabis potency. (e) Cannabis dependency. There was no significant association with any measure of cannabis use, including user status (current/ex-/never), age at first use, frequency of use (daily/weekly/less), THC content of most used cannabis type (less than 10%/more than 10%), or cannabis dependence.

In univariate survival analyses, only use of cannabis by age 15 years (HR 0.62 [95% CI: 0.32–1.18], *P* = 0.142) met our threshold (*P* < 0.2) for inclusion in subsequent multivariate analyses (Table 2, Fig. 1). In an unadjusted mixed-model Cox regression analysis, which used site as a random effect, the association was not significant (HR = 0.61 [95% CI: 0.32–1.17] *P* = 0.135). No potential confounding variables met our threshold (*P* < 0.2) for inclusion in multivariate analyses (Table S5).

### Persistence of symptoms

Among subjects for whom CAARMS follow up data were available (*n* = 209), 137 (65.6%) either still met CAARMS criteria for the CHR state or had transitioned to a full-blown psychotic disorder, and 72 (34.4%) were in symptomatic remission. In univariate analyses, two cannabis use variables met our threshold (*P* < 0.2) for inclusion in subsequent multilevel analyses: use of high potency cannabis (χ^2^ = 3.566, *P* = 0.059) and cannabis dependence (χ^2^ = 3.262, *P* = 0.071) (Table 3). In unadjusted multilevel logistic regression models, which included site as a random effect, neither of these two measures was significantly associated with persistence of psychotic symptoms (OR 0.60 [95% CI 0.14–2.26], *P* = 0.459; OR 3.15 [95% CI 1.04–11.38], *P* = 0.054). Three potentially confounding variables were identified in univariate analyses: alcohol use (*t* = 1.551, *P* = 0.123), current drug use (χ^2^ = 3.827, *P* = 0.050) and current drug dependence (*P* = 0.170, Fisher’s exact test) (Table S6). None of these improved the accuracy of the final multilevel models when added as covariates.

### Level of functioning at follow-up

In CHR subjects for whom GAF disability data were available (*n* = 215), the mean score at final follow-up was 61.5 (SD = 14.6), with an interquartile range of 50.0–73.0. GAF disability score at follow-up was significantly associated with GAF disability score at baseline (*R* = 0.329 *P* = <0.001).

In univariate analyses, two cannabis use variables met our threshold (*P* < 0.2) for inclusion in subsequent multivariate analyses: cannabis dependence (*t* = 1.630 df = 136, *P* = 0.105) and frequency of cannabis use (*F*(2,159) = 1.861, *P* = 0.159). In multilevel linear regression models, which included time of follow-up assessment as a covariate and site as a random effect, the association with cannabis dependence was not significant (estimate = −5.1 [95% CI −11.2 to 1.1], *P* = 0.105). Daily use of cannabis was significantly associated with level of functioning at follow-up compared to less than weekly use (estimate = −5.8 [95% CI −11.0 to −0.6], *P* = 0.029), and compared to less than daily use (estimate = −5.7 [95% CI −10.7 to −0.6], *P* = 0.027). However, these associations were no longer significant after adjusting for baseline GAF disability score (Table 4).

Three potentially confounding variables were identified in univariate analyses: age (*R* = −0.098, *P* = 0.153), lifetime use of other drugs (*t* = −1.692 df = 210, *P* = 0.092) and drug dependence within year to baseline (*t* = 1.728 df = 213, *P* = 0.085) (Table S7). Although adjusting for lifetime drug use improved the accuracy of the multilevel linear regression model for frequency of use (χ^2^ = 6.5771 *P* = 0.010), the association with functional outcome remained non-significant. Similarly, adjusting for lifetime drug use improved the accuracy of the multilevel linear regression model for cannabis dependence (χ^2^ = 6.3143 *P* = 0.012), but the association with functional outcome remained non-significant (Table 4).

## Discussion

Our primary hypothesis was that cannabis use in CHR subjects would be associated with an increased rate of later transition to psychosis. However, there was no significant association with any measure of cannabis use. These results are in keeping with the study by Buchy *et al*.,^26^ who followed 362 CHR subjects for 2 years and found no association between either the frequency of use, or the age at first use of cannabis and transition to psychosis. Conversely, Valmaggia *et al*.^25^ in a study of 182 CHR subjects reported that both frequent use and use before age 15 years were linked to later onset of psychosis. 52.2% of CHR participants in that study reported using cannabis at least once per week, compared to 32.6% of CHR participants who were current more-than-weekly users in the study of Buchy *et al*.^26^ (who did not find an association between frequency of use and transition), and 47.0% of CHR participants using more than once weekly in the present study. Another study in 341 CHR individuals found an association between cannabis use and transition, but this was no longer significant after controlling for alcohol use.^42^ In the present study, alcohol use did not significantly influence the findings. Although the total number of studies that have examined the link between cannabis use in CHR individuals and transition to psychosis is still modest, meta-analyses of data from these studies have not found a significant association.^23,24,59^

The lack of an association between cannabis use and psychosis onset contrasts with data from cross-sectional studies that have examined cannabis use in patients with a psychotic disorder and controls. These suggest that initiation of use at an early age,^5–7^ frequent use,^7,10^ and the use of high-THC preparations^2,7^ are associated with an increased risk of psychosis. For example, di Forti *et al*. found that a greater proportion of patients with first episode psychosis than healthy controls had used cannabis by age 15 (FEP = 28.6% *vs*. HC = 13.7%), used more than once per week (41.4% *vs*. 14.2%) and used cannabis with estimated *≥* 10% THC (37.1% *vs*. 19.4%).^7^ In the present study, 49.2% of CHR participants had used cannabis by age 15, 47.0% used more than weekly and 76.2% used high potency cannabis. As well as having the risk of recall bias, associations found by these cross-sectional studies might be confounded by the effects of other risk factors for psychosis, such as social adversity, genetic risk, and use of other substances.^12,60^ Mendelian randomization studies, which can control for such effects, indicate a causal relationship between initiation of cannabis use and schizophrenia,^13,61^ although the effect of schizophrenia risk on cannabis initiation may be even stronger. This is consistent with a study by Power *et al*. which reported an association between genetic risk for schizophrenia and both age of initiation of cannabis use and the amount of cannabis consumed.^62^

Most CHR subjects do not develop psychosis, but these individuals may still have adverse clinical outcomes in the form of persistent symptoms and an impaired level of functioning.^63,64^ Our secondary hypotheses were that cannabis use would also influence the likelihood of these two outcomes. However, we found no evidence of significant associations between any cannabis measures and either outcome. Only one previous study,^65^ has examined the association between cannabis use and persistence of the CHR state, and this also found no association. The small number of studies examining the association between cannabis use and functional outcomes in CHR subjects have produced mixed results. A cross-sectional study by MacHielsen *et al*. found no difference in GAF scores between CHR participants with and without a cannabis use disorder.^66^ In a cross-sectional study of 731 CHR and non-CHR help-seeking individuals, Carney *et al*. found that participants who showed signs of cannabis dependence and ‘high risk’ cannabis use presented with lower social and occupational functioning.^67^ However, Auther *et al*. reported that lifetime cannabis use in 101 CHR subjects was associated with a higher level of social functioning at follow-up.^68^

The present study also compared the pattern of cannabis use in people at CHR with that in controls. We found that while most (74%) CHR subjects had used cannabis before, only around a third of cannabis users were current users at the time of presentation (compared to almost half of cannabis users being current users in the healthy control sample). These observations are consistent with data from previous studies which reported that between 43% and 55% of CHR subjects had ever used cannabis, and between 22% and 30% were current users.^23,25,26,67–73^ This suggests that a large proportion of CHR individuals have stopped using cannabis before they seek clinical help. Insight is less impaired in CHR subjects than in patients with psychosis,^74^ and it is possible that many CHR subjects stop using cannabis because they find that it exacerbates their symptoms.^25,73^ It is possible that differences in level of insight and the pattern of use could explain differences between findings in studies of cannabis use and psychosis risk in CHR populations and in patients with psychotic disorders. For example, If CHR subjects tend to discontinue cannabis use, this could reduce the influence of cannabis use on the risk of psychosis in this population.^75^

Strengths of the present study were the large size of the CHR sample and the availability of detailed information on previous and current cannabis use. Although we cannot exclude the possibility that an association between cannabis use and transition to psychosis might have been evident if the follow up period had been longer than 2 years, the great majority of transitions occur within this time-frame.^22^ The present study examined the relationship between cannabis use and transition to psychosis in a sample of people at CHR for psychosis. However, the CHR population appears to be heterogenous,^76^ and the nature of the relationship between cannabis use and psychosis risk may vary between different subgroups. People are categorized as being at CHR for psychosis because of subthreshold psychotic symptoms, but the causes of these symptoms may differ between each person.^77^ For example, some people at CHR might experience attenuated psychotic symptoms due to genetic and environmental factors other than cannabis use. In others, their symptoms may be related to cannabis use, even if this is not necessary or sufficient for the development of a psychotic disorder.^77^ As both these subgroups have an increased risk of psychosis, it may be difficult to find a difference in the incidence of psychosis when cannabis users and non-users within a CHR sample are compared. In addition, many of those who may be experiencing cannabis induced attenuated psychotic symptoms could have already stopped using cannabis before baseline assessment. One way to examine this theory would be to investigate the temporal relationship between within-subject changes in cannabis use and clinical outcomes.^78^ However, this is not possible in the present study, as follow up data on cannabis use were not available in 36% of the cohort. Moreover, in almost all of the participants who transitioned to psychosis, the follow up assessments of cannabis use were made *after* the point of transition. As a result, it is not possible to know whether longitudinal changes in cannabis occurred before or after the onset of psychosis. It was thus not possible for us to address this issue in the present dataset. Because it was also not possible to collect information on clinical outcome for the entire sample, there is a risk that subjects with adverse clinical outcomes might have been more likely to be lost to follow up. However, there were no significant socio-demographic or clinical differences between those who completed follow-up and those who did not.

The present study did not include biological measures of cannabis and other substances, and future investigations could be enhanced by collecting serial urine or blood samples to corroborate interview data. Finally, although we examined cannabis use prior to the onset of psychosis, the mean age of the participants was 22 years. Our measures of cannabis use in childhood and adolescence were therefore retrospective and might not have been accurate enough to detect associations between very early use and clinical outcomes in adulthood.

## Conclusions

There was no evidence that cannabis use in people at high risk for psychosis had a significant effect on the incidence of psychosis or other adverse clinical outcomes. These findings are not consistent with epidemiological data linking cannabis use to an increased risk of developing psychosis.

## Supplementary Material

Fig. S1

Table S1, Table S2, Table S3, Table S4, Table S5, Table S6, Table S7, Supplementary Materials

## Figures and Tables

**Table 1:** Demographic, clinical and cannabis use features of CHR and control groups

	HC (*n* = 67)	CHR (*n* = 334)	*P* value
Age, years (SD)	22.9 (4.1)	22.4 (5.0)	0.478
Male gender	34 (50.7%)	177 (53.0%)	0.737
Ethnicity	–	–	0.305
White	42 (62.7%)	239 (71.6%)	
Black	10 (14.9%)	33 (9.9%)	
Other	15 (22.4%)	62 (18.6%)	
Taking antipsychotic medication	0	32 (10.3%)	**0.010**
Current tobacco use	18 (27.7%)	180 (55.4%)	**<0.001**
Other substance use (ever)	25 (37.3%)	125 (37.5%)	0.972
Cannabis use status	–	–	0.064
Current user	18 (26.9%)	90 (26.9%)	
Ex-user	23 (34.3%)	158 (47.3%)	
Never	26 (38.8%)	86 (25.7%)	
First cannabis use ≤15 years	15 (36.6%)	117 (49.2%)	0.136
Frequency of cannabis use	–	–	**0.005**
Daily	3 (7.7%)	78 (33.1%)	
More than once weekly	6 (15.4%)	33 (14.0%)	
Less than once weekly	30 (76.9%)	125 (53.0%)	
High (>10%) THC content of most used cannabis	14 (43.8%)	125 (76.2%)	**<0.001**
type			
Cannabis dependence	3 (8.6%)	36 (17.9%)	0.170

Abbreviations: CHR, clinical high risk; HC, healthy control.

Note: *P* values for *χ^2^* tests. Data as mean (SD) or *n* (%). Significant (<0.05) *P* values in bold.

**Table 2:** Relationship between cannabis use and time to transition to psychosis

	Crude HR (95% CI)	*P* value	Fully adjusted HR (95% CI)	*P* value
Cannabis use status				
Current user	1.04 (0.49−2.22)	0.914	–	–
Ex-user	1.14 (0.59−2.20)	0.707	–	–
Never	1 (ref.)	–	–	–
Age first used cannabis				
≤15 years	0.62 (0.32−1.18)	0.142	0.61 (0.32−1.17)	0.135
>15 years	1 (ref.)	–	1 (ref.)	–
Frequency of cannabis use				
Daily	0.95 (0.46−1.93)	0.876	–	–
More than once weekly	1.55 (0.68−3.51)	0.297	–	–
Less than once weekly	1 (ref.)	–	–	–
THC content of most used cannabis type				
High (>10% THC)	0.83 (0.35−1.98)	0.679	–	–
Low (<10% THC)	1 (ref.)	–	–	–
Cannabis dependence				
Dependent	1.42 (0.41−1.42)	0.383	–	–
Not dependent	1 (ref.)	–	–	–

*Note*: Crude HRs are unadjusted for confounders whereas fully adjusted HRs are adjusted for site as a random effect. Only variables with crude HR *P* < 0.2 added to adjusted, multilevel model, to reduce error from multiple testing.

Abbreviations: HR, Hazard Ratio; ref., reference category.

**Table 3:** Relationship between cannabis use and persistence of symptoms *vs*. symptomatic remission

	CHR-R (*n =* 72)	CHR-NR (*n* = 137)	*P* value	Fully adjusted OR (95% CI)	*P* value
Cannabis use status			0.304		
Current user	16 (22.2%)	44 (32.1%)		–	–
Ex-user	37 (51.4%)	64 (46.7%)		–	–
Never	19 (26.4%)	29 (21.2%)		–	–
Age first used cannabis			0.866		
≤15 years	26 (49.1%)	53 (50.5%)		–	–
>15 years	27 (50.9%)	52 (49.5%)		–	–
Frequency of cannabis use			0.245		
Daily	12 (23.5%)	39 (36.8%)		–	–
More than once weekly	8 (15.7%)	15 (14.2%)		–	–
Less than once weekly	31 (60.8%)	52 (49.1%)		–	–
THC content of most used cannabis type			0.059		
High (>10% THC)	36 (90.0%)	48 (75.0%)		0.60 (0.14−2.26)	0.459
Low (<10% THC)	4 (10.0%)	16(25.0%)		1 (ref.)	–
Cannabis dependence			0.071		
Dependent	5 (10.9%)	21 (23.9%)		3.150 (1.04−11.38)	0.054
Not dependent	41 (89.1%)	67 (76.1%)		1 (ref.)	–

*Note*: Fully adjusted ORs are adjusted for site as a random effect. Only variables with *P* < 0.2 in χ^2^ tests added to adjusted, multilevel model, to reduce error from multiple testing.

Abbreviations: CHR-R, clinical high risk remission subgroup; CHR-NR, clinical high risk persistent symptoms subgroup; OR, odds ratio; ref., reference category.

**Table 4:** Relationship between cannabis use and functional outcome

	GAF score at follow up (95% CI)	*P* value	Crude estimate (95% CI)	*P* value	Fully adjusted estimate (95% CI)	*P* value
Cannabis use status		0.346				
Current user	59.3 (55.5–63.1)		–	–	–	–
Ex-user	62.6 (59.7–65.5)		–	–	–	–
Never	62.0 (58.2–65.8)		–	–	–	–
Age first used cannabis		0.496				
≤15 years	61.0 (57.4–64.5)		–	–	–	–
>15 years	62.6 (59.6–65.5)		–	–	–	–
Frequency of cannabis use		0.159				
Daily	57.9 (53.4–62.5)		–3.3 (–8.4–1.9)	0.213	–4.4 (–9.5–0.8)	0.094
More than once weekly	62.3 (56.0–68.7)		–1.2 (–7.1–4.8)	0.698	–1.2 (–7.0–4.6)	0.674
Less than once weekly	63.1 (60.1–66.2)		0 (ref.)	–	0 (ref.)	–
THC content of most used cannabis type		0.548				
High (>10% THC)	63.2 (60.2–66.2)		–	–	–	–
Low (<10% THC)	60.8 (51.0–70.5)		–	–	–	–
Cannabis dependence		0.105				
Dependent	57.0 (50.2–63.8)		–3.5 (–9.3–2.3)	0.240	–5.3 (–11.2–0.62)	0.079
Not dependent	62.2 (59.6–64.8)		0 (ref.)	–	0 (ref.)	–

*Note*: GAF score at follow up given as mean (95% confidence interval), where higher scores represent higher levels of functioning. Estimates represent difference in mean GAF scores from reference group. Crude estimates are adjusted for baseline GAF score, days from baseline to final GAF assessment and for site as a random effect. Fully adjusted estimates are additionally adjusted for lifetime drug use. Only variables with *P* < 0.2 in *t* test or ANOVA added to adjusted, multilevel models, to reduce error from multiple testing. Abbreviations: GAF, global assessment of functioning score; ref., reference category.
