# Supplementary figures and images for: Influence of cannabis use on incidence of psychosis in people at clinical high risk
Source: Psychiatry Clin Neurosci. Author manuscript; Available in PMC 2024 Jan 29. (PMC7615575; doi:10.1111/pcn.13555)

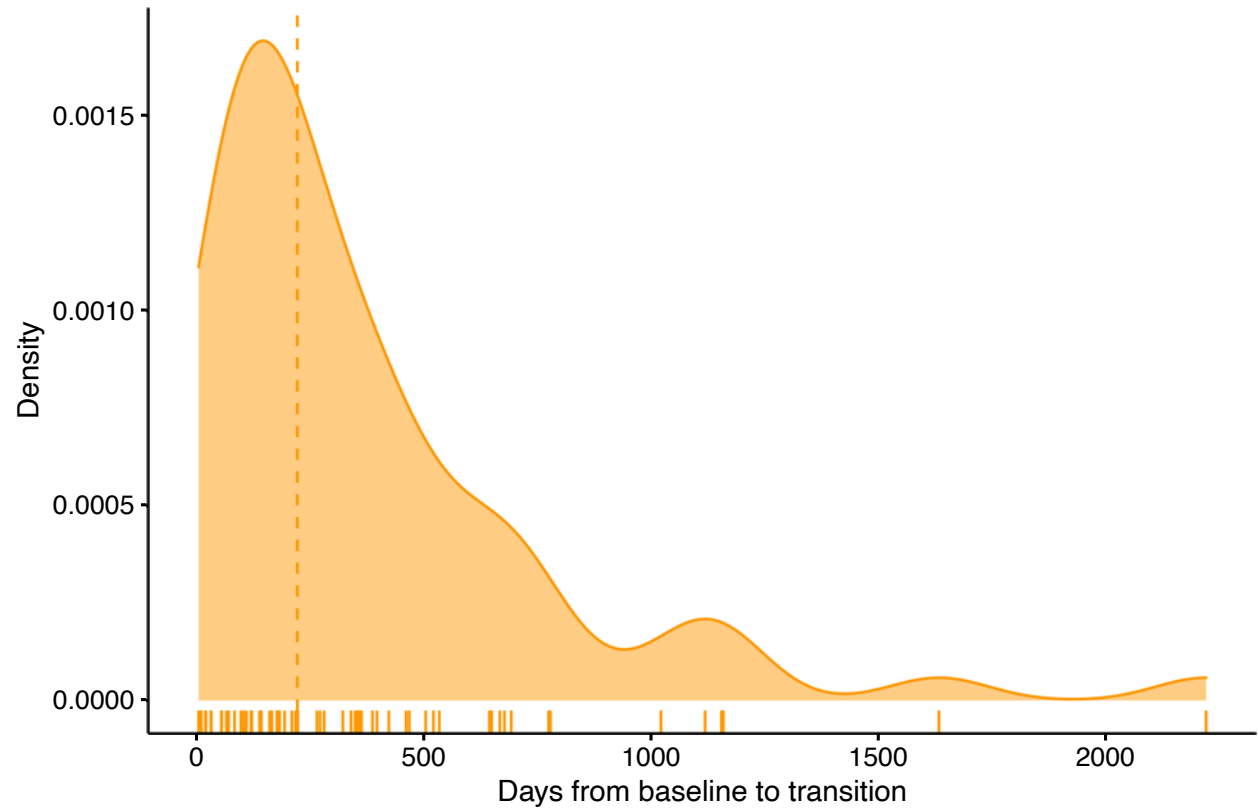

Supplement: Fig. S1 [file EMS193516-supplement-Fig__S1.pdf]
