# Supplementary material for: Influence of cannabis use on incidence of psychosis in people at clinical high risk
Source: Psychiatry Clin Neurosci. Author manuscript; Available in PMC 2024 Jan 29. (PMC7615575; doi:10.1111/pcn.13555)
Supplement: Table S1, Table S2, Table S3, Table S4, Table S5, Table S6, Table S7, Supplementary Materials [file EMS193516-supplement-Table_S1__Table_S2__Table_S3__Table_S4__Table_S5__Table_S6__Table_S7___Supplementary_Materials.docx]

# Supplementary Material

Table of Contents

[EU-GEI High Risk Study Group 2](#_Toc131683758)

[AFFILIATIONS OF GROUP AUTHOR 2](#_Toc131683759)

[Methods 4](#_Toc131683760)

[Multilevel linear regression parameter estimates 4](#_Toc131683761)

[THC content of cannabis variable 4](#_Toc131683762)

[**Table S1.** Classification of cannabis products as high (>10%) or low (<10%) THC content. 5](#_Toc131683763)

[Results 6](#_Toc131683764)

[**Figure S1.** Density plot of time to transition to psychosis. 6](#_Toc131683765)

[**Table S2**. Relationship between cannabis use and CHR status, restricted to four sites. 7](#_Toc131683766)

[**Table S3.** Subgroup analyses assessing CHR sample characteristics and missingness of follow-up data. 8](#_Toc131683767)

[**Table S4.** Demographic and clinical features of CHR participants by cannabis use status. 9](#_Toc131683768)

[**Table S5.** Demographic and clinical features of transitioned and non-transitioned CHR participants. 10](#_Toc131683769)

[**Table S6.** Demographic and clinical features of CHR participants with and without persistent symptoms at last follow-up. 11](#_Toc131683770)

[**Table S7.** Relationship between demographic and clinical features of CHR participants and GAF score at follow-up. 12](#_Toc131683771)

[References 13](#_Toc131683772)

# EU-GEI High Risk Study Group

Philip McGuire ^1^, Lucia R. Valmaggia ^2^, Matthew J. Kempton ^1^, Maria Calem ^1^, Stefania Tognin ^1^, Gemma Modinos ^1^, Lieuwe de Haan ^3,4^, Mark van der Gaag ^5,6^, Eva Velthorst ^3,7^, Tamar C. Kraan ^3^, Daniella S. van Dam ^3^, Nadine Burger ^6^, Barnaby Nelson ^8,9^, Patrick McGorry ^8,9^, G Paul Amminger ^8,9^, Christos Pantelis ^10^, Athena Politis ^8,9^, Joanne Goodall ^8,9^, Anita Riecher-Rössler ^11^, Stefan Borgwardt ^11^, Erich Studerus ^12^, Rodrigo Bressan ^13^, Ary Gadelha ^13^, Elisa Brietzke ^14^, Graccielle Asevedo ^13^, Elson Asevedo ^13^, Andre Zugman ^13^, Neus Barrantes-Vidal ^15^, Tecelli Domínguez-Martínez ^16^, Anna Racioppi ^17^, Thomas R. Kwapil ^18^, Manel Monsonet ^17^, Lídia Hinojosa ^17^, Mathilde Kazes ^19^, Claire Daban ^19^, Julie Bourgin ^19^, Olivier Gay ^19^, Célia Mam-Lam-Fook ^19^, Marie-Odile Krebs ^19^, Dorte Nordholm ^20^, Lasse Randers ^20^, Kristine Krakauer ^20^, Louise Glenthøj ^20^, Birte Glenthøj ^21^, Merete Nordentoft ^20^, Stephan Ruhrmann ^22^, Dominika Gebhard ^22^, Julia Arnhold ^23^, Joachim Klosterkötter ^22^, Gabriele Sachs ^24^, Iris Lasser ^24^, Bernadette Winklbaur ^24^, Philippe A Delespaul ^25,26^, Bart P. Rutten ^25^, Jim van Os ^1,25^

## AFFILIATIONS OF GROUP AUTHOR

1. Department of Psychosis Studies, Institute of Psychiatry, Psychology & Neuroscience, King's College London, De Crespigny Park, Denmark 458 Hill, London, United Kingdom SE5 8AF.
2. Department of Psychology, Institute of Psychiatry, Psychology & Neuroscience, King's College London, De Crespigny Park, Denmark Hill, 456 London, United Kingdom SE5 8AF.
3. Amsterdam UMC, Department Early Psychosis, Meibergdreef 5, 1105 AZ Amsterdam, The Netherlands.
4. Arkin Amsterdam
5. VU University, Faculty of Behavioural and Movement Sciences, Department of Clinical Psychology and EMGO+ Institute for Health and Care Research, van der Boechorststraat 1, 1081 BT Amsterdam, The Netherlands.
6. Parnassia Psychiatric Institute, Department of Psychosis Research, Zoutkeetsingel 40, 2512 HN The Hague, The Netherlands.
7. Icahn School of Medicine at Mount Sinai, department of Psychiatry, 1425 Madison Ave, New York, NY 10029.
8. Centre for Youth Mental Health, University of Melbourne, Parkville, Victoria 485 3052, Australia.
9. Orygen, 35 Poplar Rd, Parkville 3052, Victoria, Australia
10. Melbourne Neuropsychiatry Centre, University of Melbourne & Melbourne Health, Carlton South, Vic, Australia
11. Faculty of Medicine, University of Basel, Switzerland.
12. Department of Psychology, Division of Personality and Developmental Psychology, University of Basel, Switzerland
13. LiNC - Lab Interdisciplinar Neurociências Clínicas, Depto Psiquiatria, Escola Paulista de Medicina, Universidade Federal de São Paulo – UNIFESP.
14. Depto Psiquiatria, Escola Paulista de Medicina, Universidade Federal de São Paulo – UNIFESP.
15. Departament de Psicologia Clínica i de la Salut (Universitat Autònoma de Barcelona), Fundació Sanitària Sant Pere Claver (Spain), Spanish Mental Health Research Network (CIBERSAM).
16. CONACYT-Dirección de Investigaciones Epidemiológicas y Psicosociales, Instituto Nacional de Psiquiatría Ramón de la Fuente Muñiz (México).
17. Departament de Psicologia Clínica i de la Salut (Universitat Autònoma de Barcelona).
18. Department of Psychology, University of Illinois at Urbana-Champaign (USA).
19. University Paris Descartes, Hôpital Sainte-Anne, C’JAAD, Service Hospitalo-Universitaire, Inserm U894, Institut de Psychiatrie (CNRS 3557) Paris, France
20. Mental Health Center Copenhagen and Center for Clinical Intervention and Neuropsychiatric Schizophrenia Research, CINS, Mental Health Center Glostrup, Mental Health Services in the Capital Region of Copenhagen, University of Copenhagen.
21. Centre for Neuropsychiatric Schizophrenia Research (CNSR) & Centre for Clinical Intervention and Neuropsychiatric Schizophrenia Research (CINS), Mental Health Centre Glostrup, University of Copenhagen, Glostrup, Denmark
22. Department of Psychiatry and Psychotherapy, Faculty of Medicine and University Hospital, University of Cologne, Cologne, Germany.
23. Psyberlin, Berlin, Germany.
24. Medical University of Vienna, Department of Psychiatry and Psychotherapy.
25. Department of Psychiatry and Neuropsychology, School for Mental Health and Neuroscience, Maastricht University Medical Centre, P.O. Box 616, 6200 MD 464 Maastricht, The Netherlands
26. Mondriaan Mental health Trust, P.O. Box 4436 CX Heerlen, The Netherlands

# Methods

## Multilevel linear regression parameter estimates

Fixed effect parameter estimates are interpreted the same way as one would interpret estimates from a traditional ordinary least squares linear regression. For instance, for a categorical predictor with a coefficient of 1.0, the mean GAF score of the predictor group of is 1.0 points higher than the mean GAF score of the reference group.

## THC content of cannabis variable

Participants were asked to name in their own language the type of cannabis they mostly used during their period of use. Types named included hash (cannabis resin/solid), imported herbal cannabis, home-grown skunk/ sensimilla / super skunk, and the Dutch geïmporteerde wiet (imported herbal cannabis), Nederwiet (Dutch herbal cannabis), geïmporteerde hasj (imported cannabis resin) and Nederhasj (﻿cannabis resin made of Nederwiet). Other named types or answers that could not be clearly grouped, such as “all of them” or “don’t know”, were excluded from this analysis.

The THC content variable was created using a cut off of 10% THC, consistent with previous research.^1,2^ Data published by the European Monitoring Centre for Drugs and Drug Addiction (EMCDDA) reports^3–9^ as well as other national reports on cannabis potency^10–19^ was stratified according to country and cannabis type and used to classify the types of cannabis named by participants as either high (>10%) or low (<10%) THC content (see Table S1 for more details).

## **Table S1.** Classification of cannabis products as high (>10%) or low (<10%) THC content.

|  | **Named types of cannabis products** | | | | | | |
| --- | --- | --- | --- | --- | --- | --- | --- |
| **Site** | Hash (cannabis resin/solid) | Imported herbal cannabis | Home-grown skunk/ Sensimilla/  Super skunk | Geïmporteerde hasj | Nederhasj | Geïmporteerde wiet | Nederwiet |
| London | Low | Low | High |  |  |  |  |
| Vienna | Low | Low |  |  |  |  |  |
| Basel | High | Low | High |  |  |  |  |
| Cologne | Low | High |  |  |  |  |  |
| Melbourne | High |  | High |  |  |  |  |
| Copenhagen | High | High |  |  |  |  |  |
| Paris | High | High |  |  |  |  |  |
| Barcelona | High | High | High |  |  |  |  |
| Sao Paulo |  | Low |  |  |  |  |  |
| Amsterdam |  |  |  | High | High | Low | High |
| Den Haag |  |  |  | High | High | Low | High |
| High = high estimated THC content (>10%); Low = low estimated THC content (<10%).  Geïmporteerde hasj = Dutch imported cannabis resin; Nederhasj = Dutch cannabis resin made of Nederwiet; Geïmporteerde wiet = Dutch imported herbal cannabis; Nederwiet = Dutch herbal cannabis.  Blank cells indicate that no participants from the site named that type of cannabis product. | | | | | | | |

# Results

## **Figure S1.** Density plot of time to transition to psychosis.

Dashed lines mark median time to transition (223 days) and 2-years post-baseline respectively.

## **Table S2**. Relationship between cannabis use and CHR status, restricted to four sites.

|  | HC (n=67) | CHR (n=210) | p value |
| --- | --- | --- | --- |
| Cannabis use | -- | -- | **0.010** |
| *Current user* | 18 (26.9%) | 61 (29.0%) | -- |
| *Ex-user* | 23 (34.3%) | 105 (50.0%) | -- |
| *Never* | 26 (38.8%) | 44 (21.0%) | -- |
| Frequency of cannabis use | -- | -- | **0.011** |
| *Daily* | 3 (7.7%) | 51 (31.1%) | -- |
| *More than once weekly* | 6 (15.4%) | 22 (13.4%) | -- |
| *Less than once weekly* | 30 (76.9%) | 91 (55.5%) | -- |
| THC content of most used cannabis type | -- | -- | **<0.001** |
| *High potency* | 14 (43.8%) | 85 (76.6%) | -- |
| *Low potency* | 18 (56.3%) | 26 (23.4%) | -- |
| Age of first cannabis use | -- | -- | 0.141 |
| *≤15 years* | 15 (36.6%) | 82 (49.4%) | -- |
| *>15 years* | 26 (63.4%) | 84 (50.6%) | -- |
| Cannabis dependence | -- | -- | 0.387 |
| *Yes* | 3 (8.6%) | 19 (14.1%) | -- |
| *No* | 32 (91.4%) | 116 (85.9%) | -- |
| Abbreviations: CHR, clinical high risk; HC, healthy control.  P values for $\chi$^2^ tests. Data as mean (SD) or n (%). Significant (<0.05) p values in bold. | | | |

## **Table S3.** Subgroup analyses assessing CHR sample characteristics and missingness of follow-up data.

|  | Followed-up (n=259) | No follow-up (n=75) | p value |
| --- | --- | --- | --- |
| Age, years (SD) | 22.6 (4.9) | 21.8 (5.2) | 0.262 |
| Male gender | 142 (54.8%) | 35 (46.7%) | 0.212 |
| Ethnicity | -- | -- | 0.167 |
| *White* | 186 (71.8%) | 53 (70.7% | -- |
| *Black* | 29 (11.2%) | 4 (5.3%) | -- |
| *Other* | 44 (17.0%) | 18 (24.0%) | -- |
| Taking antipsychotic medication | 23 (9.4%) | 9 (13.8%) | 0.294 |
| Tobacco use, daily | 144 (57.6%) | 34 (46.6%) | 0.096 |
| Alcohol, drinks per week (SD) | 5.4 (9.5) | 6.3 (12.9) | 0.509 |
| Other substance use, ever | 101 (39.5%) | 23 (30.7%) | 0.167 |
| Other substance use, past year | 76 (32.2%) | 21 (28.4%) | 0.536 |
| Other substance dependence, ever | 21 (8.1%) | 7 (9.3%) | 0.736 |
| Other substance dependence, past year | 11 (4.2%) | 4 (5.3%) | 0.689 |
| P values for $\chi$^2^ tests. Data as mean (SD) or n (%). | | | |

## **Table S4.** Demographic and clinical features of CHR participants by cannabis use status.

|  | Never used (n=86) | Past user (n=158) | Current user (n=90) | p value |
| --- | --- | --- | --- | --- |
| Age, years | 20.5 (4.7) | 22.9 (4.5)^a^ | 23.3 (5.5)^a^ | **<0.001** |
| Male gender | 38 (44.2%) | 81 (51.3%) | 58 (64.4%)^a^ | **0.022** |
| Ethnicity | -- | -- |  | 0.175 |
| *White* | 60 (69.8%) | 113 (71.5%) | 66 (73.3%) |  |
| *Black* | 5 (5.8%) | 21 (13.3%) | 7 (7.8%) |  |
| *Other* | 21 (24.4%) | 24 (15.2%) | 17 (18.9%) |  |
| Antipsychotic use | 8 (10.5%) | 15 (9.9%) | 9 (10.8%) | 0.974 |
| Tobacco use, daily | 9 (10.8%) | 99 (64.3%)^a^ | 70 (81.4%)^a, b^ | **<0.001** |
| Alcohol, drinks per week | 1.7 (5.1) | 6.4 (12.3)^a^ | 8.1 (9.4)^a^ | **<0.001** |
| Other substance use, ever | 3 (3.5%) | 66 (42.0%)^a^ | 55 (61.8%)^a, b^ | **<0.001** |
| Other substance use, past year | 2 (2.4%) | 51 (34.9%)^a^ | 44 (55.0%)^a, b^ | **<0.001** |
| Other substance dependence, ever | 0 (0%) | 1 (8.9%)^a^ | 14 (15.6%)^a, b^ | **<0.001** |
| Other substance dependence, past year | 0 (0%) | 6 (3.8%) | 9 (10.0%)^a^ | **0.004** |
| GAF disability score | 56.2 (14.2) | 55.9 (12.1) | 53.7 (10.7) | 0.393 |
| CAARMS positive symptom score | 35.0 (19.8) | 37.2 (20.8) | 38.7 (17.8) | 0.332 |
| CAARMS negative symptom score | 27.9 (20.4) | 30.3 (17.8) | 29.4 (17.8) | 0.359 |
| Abbreviations: CAARMS, Comprehensive Assessment of At-Risk Mental State; GAF, Global Assessment of Functioning.  P values for $\chi$^2^, Fisher-Freeman-Halton Exact, one-way ANOVA or Kruskal-Wallis tests. Data as mean (SD) or n (%). Significant (<0.05) p values are presented in bold.  ^a^ Significantly different from never used group, after Bonferroni correction for multiple comparisons.  ^b^ Significantly different from past user group, after Bonferroni correction for multiple comparisons. | | | | |

## **Table S5.** Demographic and clinical features of transitioned and non-transitioned CHR participants.

|  | Hazard Ratio (95% CI) | P value |
| --- | --- | --- |
| Age, years | 1.01 (0.95 – 1.06) | 0.836 |
| Gender | -- |  |
| *Male* | 1.10 (0.64 – 1.88) | 0.729 |
| *Female* | *1 (ref.)* | -- |
| Ethnicity | -- |  |
| *White* | *1 (ref.)* | -- |
| *Black* | 1.36 (0.61 – 3.04) | 0.456 |
| *Other* | 0.90 (0.43 – 1.86) | 0.771 |
| Taking antipsychotic medication | -- |  |
| *Yes* | 2.38 (1.19 – 4.76) | **0.015** |
| *No* | *1 (ref.)* | -- |
| Tobacco use, daily | -- |  |
| *Yes* | 0.83 (0.48 – 1.44) | 0.509 |
| *No* | *1 (ref.)* | -- |
| Alcohol, drinks per week | 1.00 (0.98 – 1.03) | 0.846 |
| Other substance use, ever | -- |  |
| *Yes* | 1.12 (0.64 – 1.94) | 0.695 |
| *No* | *1 (ref.)* | -- |
| Other substance use, past year | -- |  |
| *Yes* | 1.14 (0.64 – 2.05) | 0.653 |
| *No* | *1 (ref.)* | -- |
| Other substance dependence, ever | -- |  |
| *Yes* | 1.13 (0.45 – 2.84) | 0.791 |
| *No* | *1 (ref.)* | -- |
| Other substance dependence, past year | -- |  |
| *Yes* | 1.30 (0.41 – 4.16) | 0.661 |
| *No* | *1 (ref.)* | -- |
| Abbreviations: ref., reference category.  P values for Cox regression analyses. Significant (<0.05) p values in bold. | | |

## **Table S6.** Demographic and clinical features of CHR participants with and without persistent symptoms at last follow-up.

|  | CHR-R (n=72) | CHR-NR (n=137) | p value |
| --- | --- | --- | --- |
| Age, years | 22.6 (4.7) | 23.1 (5.3) | 0.447 |
| Male gender | 35 (48.6%) | 78 (56.9%) | 0.251 |
| Ethnicity | -- | -- | 0.838 |
| *White* | 52 (72.2%) | 97 (70.8%) | -- |
| *Black* | 7 (9.7%) | 17 (12.2%) | -- |
| *Other* | 13 (18.1%) | 23 (16.8%) | -- |
| Taking antipsychotic medication | 5 (7.5%) | 14 (10.7%) | 0.466 |
| Tobacco use, daily | 36 (50%) | 77 (56.2%) | 0.253 |
| Alcohol, drinks per week | 4.0 (10.5) | 6.3 (9.4) | 0.123 |
| Other substance use, ever | 26 (36.1%) | 59 (43.1%) | 0.310 |
| Other substance use, past year | 15 (23.8%) | 49 (38.0%) | 0.050 |
| Other substance dependence, ever | 6 (8.3%) | 14 (10.2%) | 0.660 |
| Other substance dependence, past year | 1 (1.4%) | 9 (6.6%) | 0.170 |
| Abbreviations: CHR-R, clinical high risk remission subgroup; CHR-NR, clinical high risk persistent symptoms subgroup.  P values for $\chi$^2^ or Fisher's Exact test. Data as mean (SD) or n (%). | | | |

## **Table S7.** Relationship between demographic and clinical features of CHR participants and GAF score at follow-up.

|  | GAF at follow up | P value |
| --- | --- | --- |
| Age, years | R= -0.098 | 0.153 |
| Gender | -- | 0.339 |
| *Male* | 60.7 (57.9 – 63.4) | -- |
| *Female* | 62.6 (59.8 – 65.4) | -- |
| Ethnicity | -- | 0.209 |
| *White* | 62.6 (60.2 – 64.9) | -- |
| *Black* | 57.4 (50.1 – 64.8) | -- |
| *Other* | 59.5 (55.4 – 63.7) | -- |
| Taking antipsychotic medication | -- | 0.179 |
| *Yes* | 56.8 (49.1 – 64.5) | -- |
| *No* | 61.8 (59.6 – 64.0) | -- |
| Tobacco use, daily | -- | 0.922 |
| *Yes* | 61.6 (58.9 – 64.3) | -- |
| *No* | 61.4 (58.4 – 64.4) | -- |
| Alcohol, drinks per week | R= -0.058 | 0.415 |
| Other substance use, ever | -- | 0.092 |
| *Yes* | 63.6 (60.5 – 66.6) | -- |
| *No* | 60.2 (57.6 – 62.8) | -- |
| Other substance use, past year | -- | 0.243 |
| *Yes* | 63.3 (60.0 – 66.8) | -- |
| *No* | 60.8 (58.2 – 63.3) | -- |
| Other substance dependence, ever | -- | 0.428 |
| *Yes* | 59.0 (51.7 – 66.3) | -- |
| *No* | 61.8 (59.7 – 63.8) | -- |
| Other substance dependence, past year | -- | 0.085 |
| *Yes* | 53.8 (45.9 – 61.7) | -- |
| *No* | 61.9 (59.9 – 63.9) | -- |
| Abbreviations: Global Assessment of Functioning score.  Data are Spearman’s rho (continuous variables) or mean (95% confidence interval) (categorical variables). P values for Spearman’s rank correlation, ANOVA or student’s t test analyses. | | |

# References

1. Di Forti M, Quattrone D, Freeman TP, et al. The contribution of cannabis use to variation in the incidence of psychotic disorder across Europe (EU-GEI): a multicentre case-control study. *The Lancet Psychiatry*. 2019;6(5):427-436. doi:10.1016/S2215-0366(19)30048-3

2. Hines LA, Freeman TP, Gage SH, et al. Association of High-Potency Cannabis Use With Mental Health and Substance Use in Adolescence. *JAMA Psychiatry*. 2020;77(10):1044. doi:10.1001/jamapsychiatry.2020.1035

3. European Monitoring Centre for Drugs and Drug Addiction. *European Drug Report 2016: Trends and Developments*.; 2016. doi:10.2810/04312

4. European Monitoring Centre for Drugs and Drug Addiction. Observatoire Français des Drogues et Des Toxicomanies. *France National Report (2013 Data) to the EMCDDA 2014*.; 2014.

5. European Monitoring Centre for Drugs and Drug Addiction. Österreichisches Bundesinstitut für Gesundheitswesen. *Austria National Report (2011 Data) to the EMCDDA 2012*.; 2012.

6. European Monitoring Centre for Drugs and Drug Addiction. Österreichisches Bundesinstitut für Gesundheitswesen. *Austria National Report (2012 Data) to the EMCDDA 2013*.; 2013.

7. European Monitoring Centre for Drugs and Drug Addiction. Österreichisches Bundesinstitut für Gesundheitswesen. *Austria National Report (2013 Data) to the EMCDDA 2014*. Gesundheit Österreich GmbH, Stubenring 6, 1010 Vienna, Austria; 2014.

8. European Monitoring Centre for Drugs and Drug Addiction. Spanish Ministry of Health and Consumer Affairs. *Spain National Report (2013 Data) to the EMCDDA 2014.* Madrid; 2014.

9. European Monitoring Centre for Drugs and Drug Addiction. Deutsche Beobachtungsstelle für Drogen und Drogensucht. *Germany National Report (2013 Data) to the EMCDDA 2014*.; 2014.

10. De Oliveira GL, Voloch MH, Sztulman GB, Neto ON, Yonamine M. Cannabinoid contents in cannabis products seized in São Paulo, Brazil, 2006-2007. *Forensic Toxicol*. 2008;26(1):31-35. doi:10.1007/s11419-008-0046-x

11. Hardwick S, King L. *Home Office Cannabis Potency Study 2008*.; 2008.

12. SGRM. *Swiss Forensic Chemistry Statistics THC Jul - Dec 2013*.; 2014.

13. Swift W, Wong A, Li KM, Arnold JC, McGregor IS. Analysis of Cannabis Seizures in NSW, Australia: Cannabis Potency and Cannabinoid Profile. *PLoS One*. 2013;8(7):1-9. doi:10.1371/journal.pone.0070052

14. Niesink RJM, Rigter S, Koeter MW, Brunt TM. Potency trends of Δ9-tetrahydrocannabinol, cannabidiol and cannabinol in cannabis in the Netherlands: 2005-15. *Addiction*. 2015;110(12):1941-1950. doi:10.1111/add.13082

15. OFDT (Observatoire Français des Drogues et des Toxicomaies). *Drugs, Key Data 2015*. Paris; 2015.

16. OFDT (Observatoire Français des Drogues et des Toxicomaies). *Drugs, Key Data 2017*. Paris; 2017.

17. Freeman TP, Van Der Pol P, Kuijpers W, et al. Changes in cannabis potency and first-time admissions to drug treatment: A 16-year study in the Netherlands. *Psychol Med*. 2018;48(14):2346-2352. doi:10.1017/S0033291717003877

18. Potter DJ, Hammond K, Tuffnell S, Walker C, Di Forti M. Potency of Δ9–tetrahydrocannabinol and other cannabinoids in cannabis in England in 2016: Implications for public health and pharmacology. *Drug Test Anal*. 2018;10(4):628-635. doi:10.1002/dta.2368

19. Thomsen KR, Lindholst C, Thylstrup B, et al. Changes in the composition of cannabis from 2000-2017 in Denmark: Analysis of confiscated samples of cannabis resin. *Exp Clin Psychopharmacol*. 2019;27(4):402-411. doi:10.1037/pha0000303
